# Relationships among Environment, Climate, and Longevity in China

**DOI:** 10.3390/ijerph14101195

**Published:** 2017-10-08

**Authors:** Yi Huang, Mark Rosenberg, Lingli Hou, Mengjin Hu

**Affiliations:** 1 School of Geographic Science, Nantong University, Nantong 226007, China; ntugeo@sina.com (L.H.); tinyyellowfish@sina.com (M.H.); 2 Department of Geography and Planning, Queen’s University, Kingston, ON K7L 3N6, Canada; rosenber@queensu.ca

**Keywords:** longevity, selenium, omega-3, sea fish, altitude, climate

## Abstract

Human longevity is influenced by environment and nutrition. We considered environmental and nutritional factors relating to longevity in Chinese cities. We found higher 85+/65+ distribution ratios, indicating enhanced longevity, in the coastal and southern regions of China. These areas also featured higher humidity, low standard deviation of monthly temperature, higher levels of selenium (Se) distribution in soil, and greater sea fish consumption. Moderate climate is more conducive to longevity, however, there is no significant difference in longevity between different sub-climatic types within moderate climate; the relation between humidity and longevity is not always positive, the relation between altitude and longevity is not always negative. Nutritional factors like Se and omega-3 fatty acids contained in sea fish were crucial to longevity. In contrast, the consumption of meat and freshwater fish were less related to longevity. Taken together, humidity, altitude, and per capita sea fish consumption, when evaluated via geographically weighted regression, explained 66% and 68% of longevity among Chinese individuals in 2000 and 2010, respectively. Other factors require further discussion.

## 1. Introduction

Along with individual lifestyle factors like psychology, sports, diet, et cetera, human health and longevity are directly influenced by the local environment and dietary nutrition. Environmental factors include climate (temperature, precipitation, humidity, etc.), and chemical and physical contaminants of the air, soil and water. Nutritional factors include major and trace dietary elements, vitamins, unsaturated fatty acids, and so forth. In the elderly, extreme temperatures have a significant impact on mortality [1,2,3,4,5,6], and exposure, or lack of exposure, to the climatic change often prompts long-term effects of harsh winters can contribute to differences in mortality among these individuals [7]. Hypoxia induced by living at higher altitudes affects health and disease [8]. As for nutrition, areas with low selenium (Se) soil content exhibit endemic diseases [9,10,11]. Omega-3 fatty acids are considered anti-inflammatory, anti-thrombosis, and anti-arrhythmic. They can reduce blood lipids, relax the vasculature, and prevent cancer [12,13].

These environmental and nutritional factors relate to disease, human health protection, and longevity. Environment factors vary greatly across the earth’s surface, and the amounts of trace elements and other nutritional factors are unequally distributed throughout the water and soil. Past studies examined climate [14], air pollution [15], and soil trace elements [16] and their relationship to longevity in China. Study findings revealed that, in South China, favorable climate has beneficial effects on longevity [14]; and air pollution can alter life expectancy [15]; moreover, the Se soil content has a significant, positive correlation with the longevity index in China [16]. Determining longevity is a complex process, and each factor necessitates comprehensive study. Additionally, past studies were conducted at the province level and data obtained on this large a scale tend to be less accurate. In this study, we considered environmental, nutritional, and their influences on longevity in China at the city level.

## 2. Data and Methods

### 2.1. Population

Population data were obtained from the demographic database of the fifth and sixth national population census of China, carried out in 2000 and 2010, respectively (China Statistics/Beijing Info 2000, 2010) [17,18]. We used data from 345 Chinese cities in our analyses. These data included the number of individuals aged 85 years and older (the oldest available age group; 85+), and the number of individuals aged 65 years and older (65+). We used the 85+/65+ ratio as a city-level measure of longevity, as it is less sensitive to factors such as migration and birth rates which can obscure other measures of longevity. 

**Figure 1:**
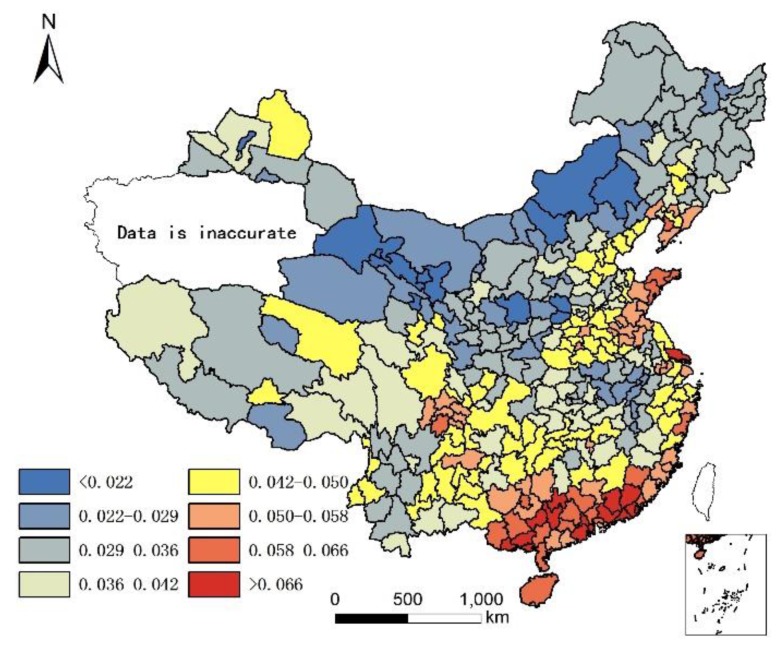
Longevity rate (85+/65+) in China in 2000.

**Figure 2:**
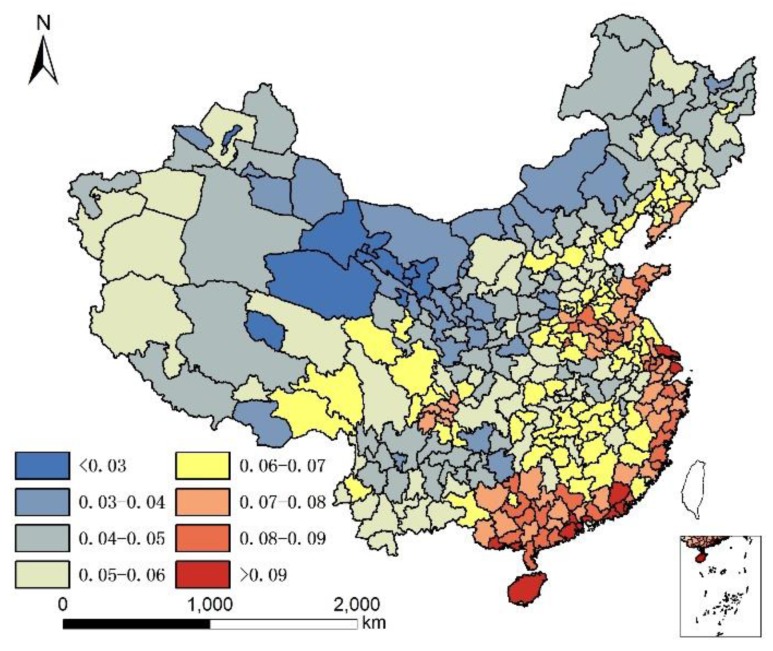
Longevity rate (85+/65+) in China in 2010.

It is acknowledged that the current population structure includes 65+ years old people who might have moved prior to where they lived in 2010. Since no data are available to take into account the migration of 65+ years old people prior to 2010, the assumption is that only the climate and local food of their present location affects their health status, although it is understood that where people lived in the past plays a role in their current health status. The geographical distribution of 85+/65+ ratios in Chinese cities in 2000 and 2010 are illustrated in Figure 1 and Figure 2.

As the population census data in south Xinjiang was not accurate [19] because investigation found the centenarians and elders count to be inaccurate as there was large gap between self-reported age and verified (actual) age [20,21]. Therefore, we deleted the data for south Xinjiang in 2000.

Two kinds of factors, potentially related to longevity, were collected for analysis: environmental and nutritional variables.

### 2.2. Environmental Factors

**Figure 3:**
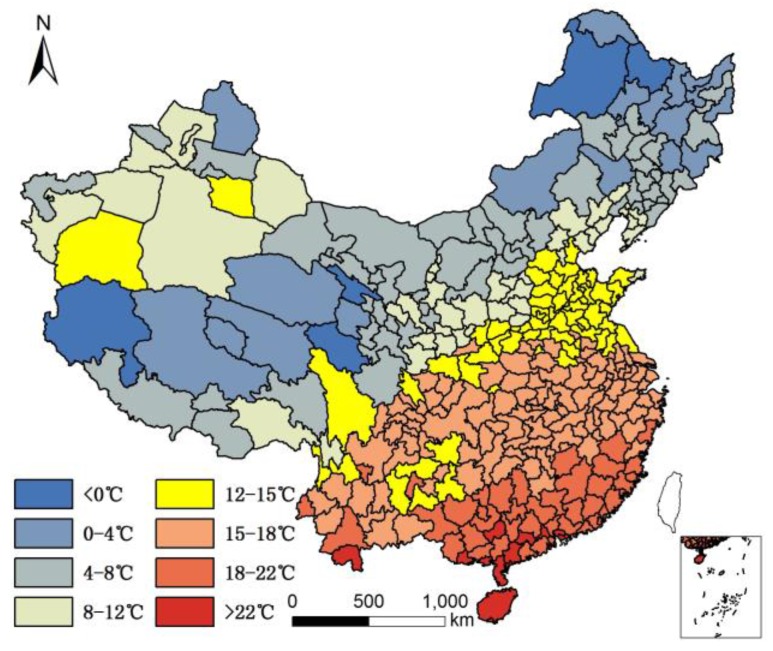
Average temperature in China.

**Figure 4:**
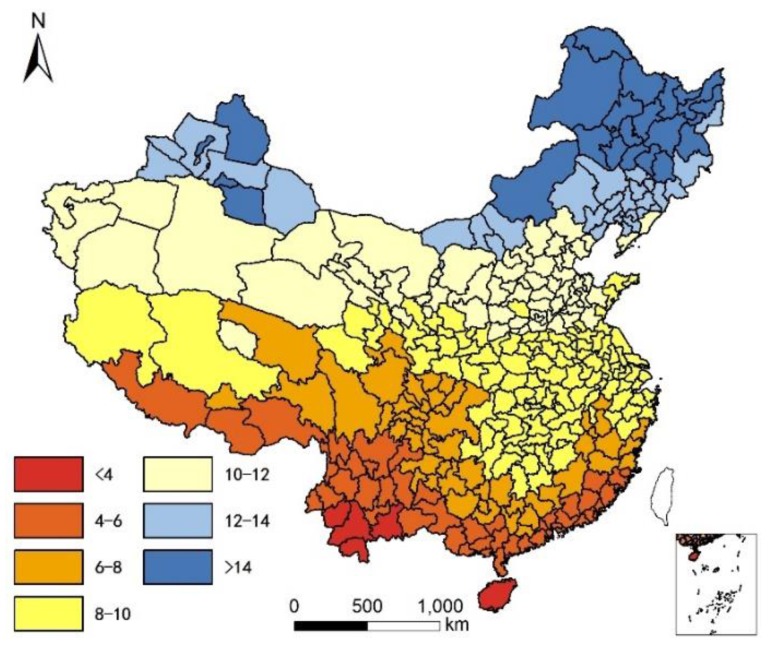
Standard deviation of monthly mean temperature in China.

We collected the standard deviation of the monthly mean temperature to exam potential relationships between extreme temperatures and longevity. Additional variables of interest included average humidity and altitude. We acquired annual average precipitation data for each city from the public meteorological service center website of the Chinese meteorological administration (www.weather.com.cn) and (www.tianqi.com). Yearly mean temperature is illustrated in Figure 3. The standard deviation of monthly mean temperature is illustrated in Figure 4. Humidity was calculated according to a classic equation formulated by de Martonne in 1926 (Equation (1)):

I = P/(T + 10)(1)

**Figure 5:**
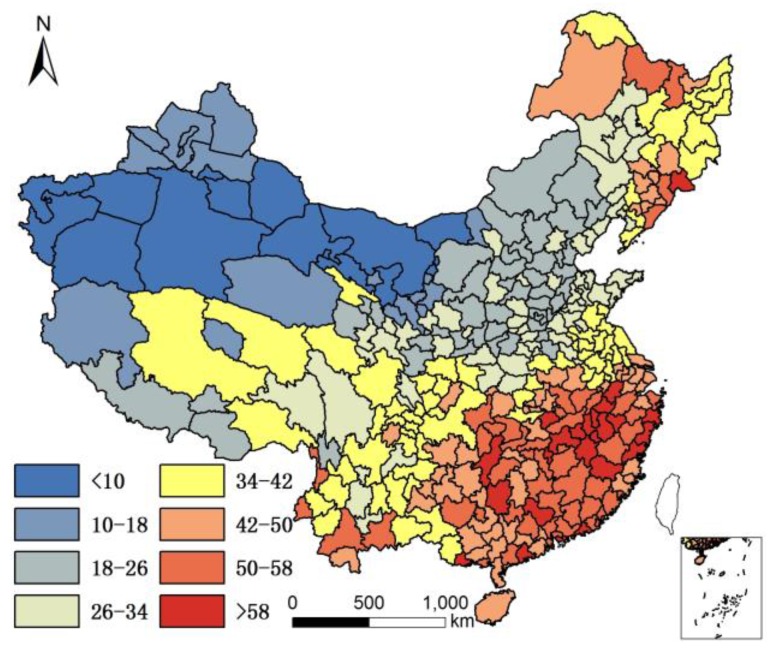
Average yearly humidity in China.

In this equation, I represents humidity; P is annual average precipitation (mm); T is annual average temperature (Centigrade). Each city’s humidity is illustrated in Figure 5 with higher values indicating higher humidity.

**Figure 6:**
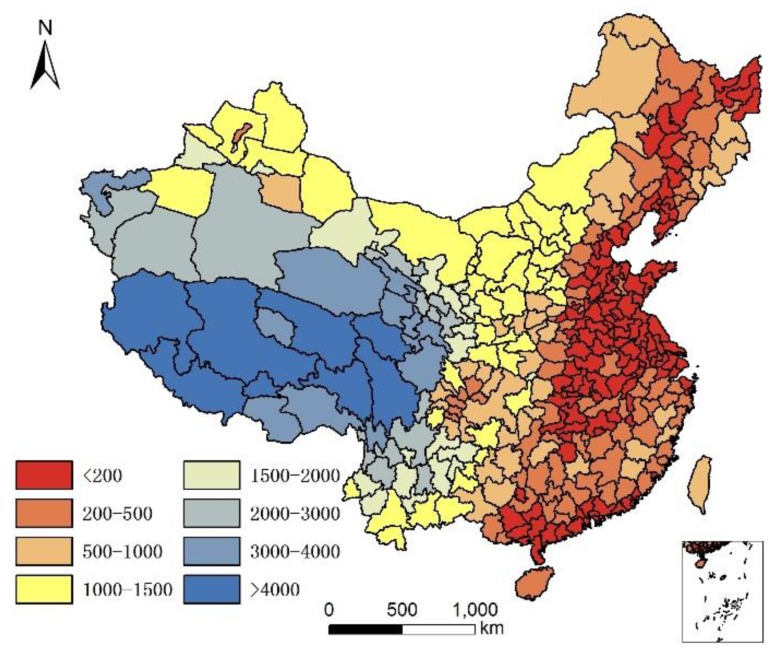
Altitude of each city in China (m).

We downloaded the digital elevation model (DEM) of China (resolution: 1 km × 1 km) from the data cloud of the Chinese Academy of Sciences (http://www.csdb.cn/), and subsequently calculated the average altitude of each city using zonal statistics analysis tools and ArcGIS software with the DEM data and map of each city. Average city altitudes are illustrated in Figure 6.

Environmental pollution is currently a serious issue in China and has been for the past 10 to 15 years. It is well known that pollution from industry, automobiles and power plants that burn coal exerts cumulative and long-term effects on human health. Most of the older people (85+) included in this analysis lived most of their lives in rural areas prior to the current pollution problems. It is also problematic to associate sources of pollution with older people at the geographic scale used in this analysis. For these reasons, no pollution variables are included in the analysis. 

### 2.3. Nutritional Factors 

**Figure 7:**
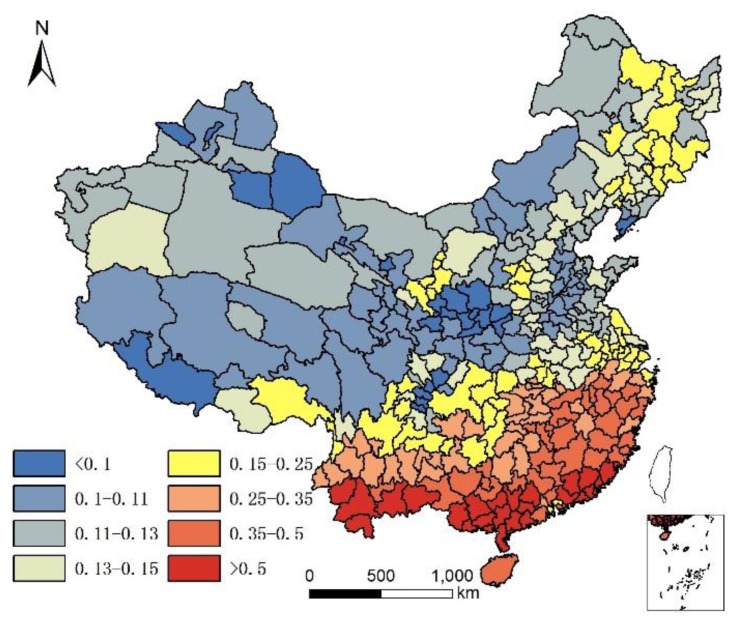
Total selenium content in soil of each city in China (mg/kg).

**Figure 8:**
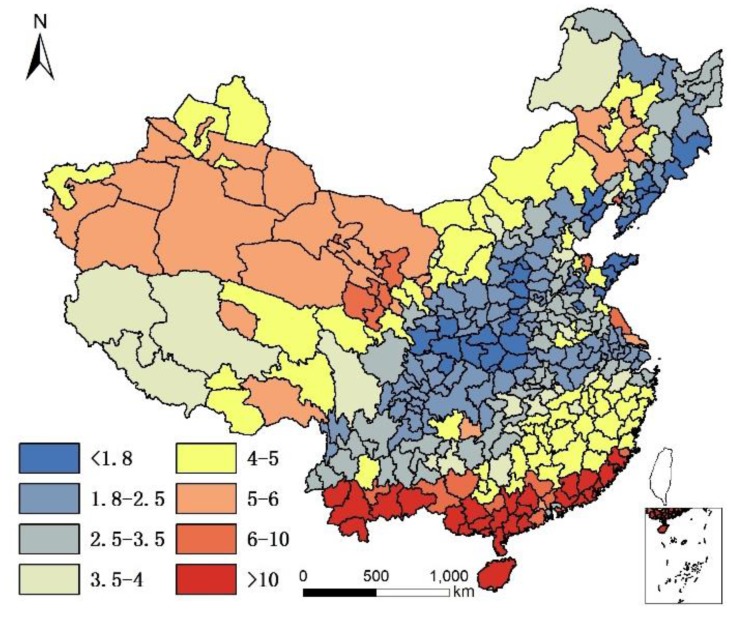
Water-soluble selenium content in soil of each city in China (ug/kg).

Studies found that the Se content in soil has a significant positive correlation with longevity. In contrast, barium (Ba) and nickel (Ni) have significant negative correlations longevity, while distributions of cadmium (Cd), cobalt (Co), chromium (Cr), copper (Cu), manganese (Mn), vanadium (V), zinc (Zn), lithium (Li), and iron (Fe) showed no significant correlations with longevity [16]. Of all the trace elements, Se appears most closely linked with longevity [22,23,24,25]. Food contributes a greater proportion of daily elemental intake than drinking water [26], and the Se content in food is positively correlates with the Se content in soil [27]. Because of this, we selected the Se content in soil to represent the relationship between longevity and trace elements. Soil Se can be considered as total Se (T-Se) and water-soluble (WS) Se (WS-Se). Soil WS-Se is a better indicator of environmental effects than T-Se [28]. In this study, both T-Se and WS-Se content of soil were considered. We collected background concentrations of T-Se and WS-Se from China’s Soil Environment Background Concentration Research (Ministry of Environmental Protection of the People’s Republic of China, China National Environmental Monitoring Centre 1990) [29] and other studies related to soil environmental background values in China [11,30]. We calculated soil T-Se and WS-Se in each city using the union and statistic tools in ArcGIS software. T-Se and WS-Se, for each city, is illustrated in Figure 7 and Figure 8.

**Figure 9:**
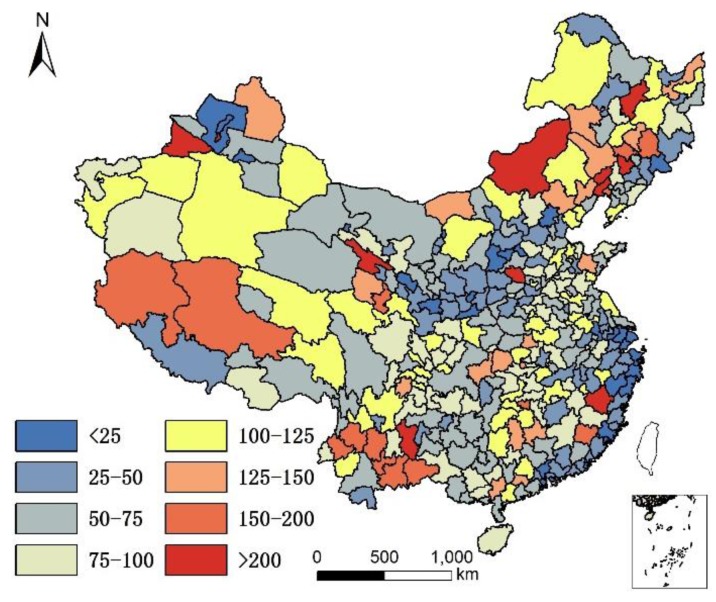
Per capita meat production of each city in China in 2016 (kg).

**Figure 10:**
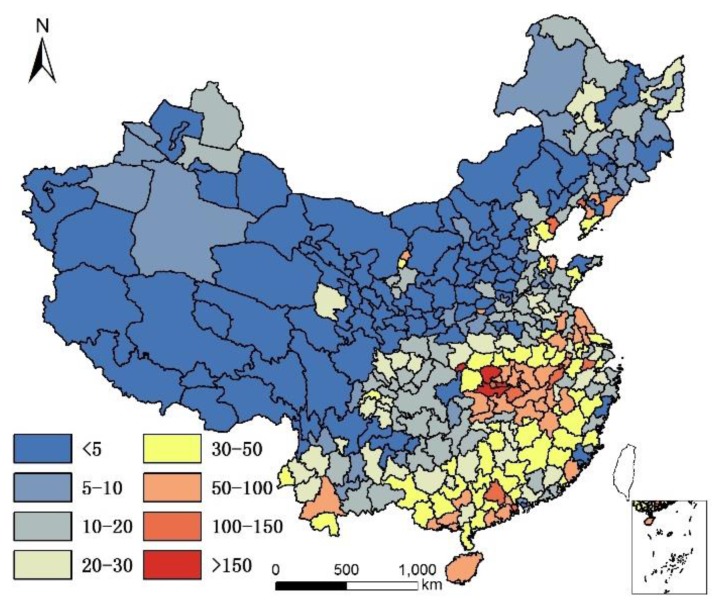
Per capita freshwater-fish production of each city in China in 2016 (kg).

**Figure 11:**
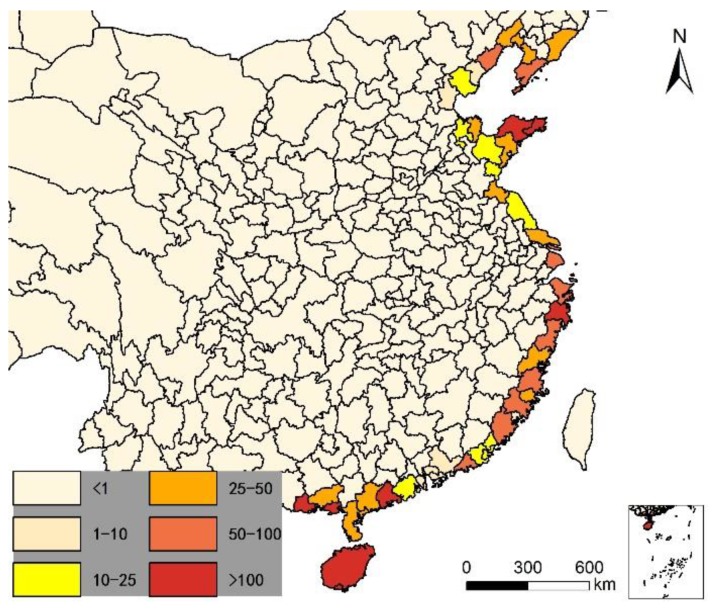
Per capita sea fishing of each city in China in 2016 (kg).

We also noted per capita meat production (Figure 9), per capita freshwater-fish production (Figure 10), and per capita seawater fish production (Figure 11 (Shanghai and Zhoushan are regarded as one fishing ground)) to examine potential relationships between omega-3 intake and longevity. These data were collected from the statistical bulletins of each city, and the 1990 (Fishery Administration Bureau of Ministry of Agriculture 1990) and 2016 (Fishery Administration Bureau of Ministry of Agriculture 2016) China Fisheries Statistics Yearbook [31,32]. Water food production in 1990 and 2015 are illustrated in Table 1.

Table 1 shows that fishing production increased slowly from 1990 to 2015, and seawater fishing and freshwater fishing increased 2.39 and 2.89 times, respectively. Because of the limitations of natural water food resources, aquaculture production increased sharply due to recent developments in aquaculture and biological technologies, seawater aquaculture and freshwater aquaculture increased 11.58 and 6.86 times, respectively. Individuals who were aged 85+ years had mainly eaten fishing productions rather than aquaculture productions throughout their lives, particularly at a young age. Consequently, seawater aquaculture production was not included in the seawater food production data in this research.

**Figure 12:**
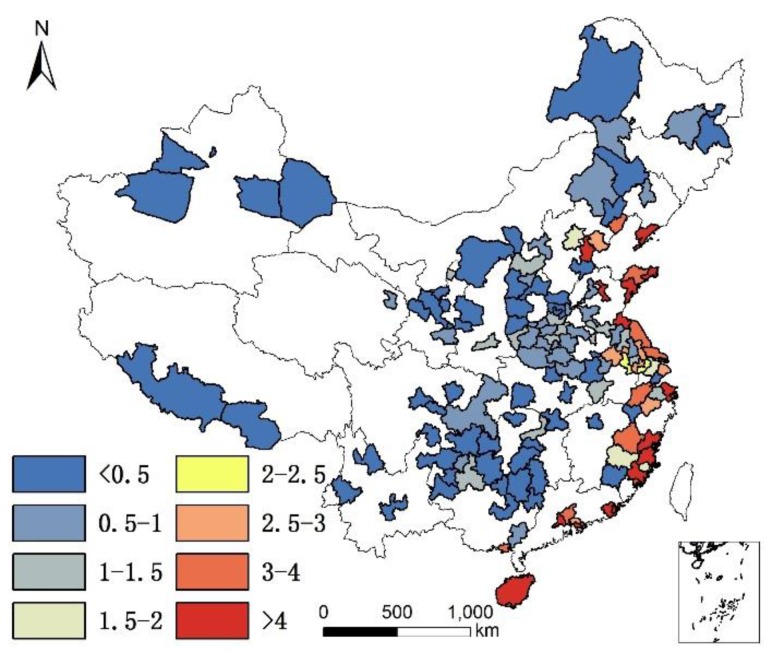
Sea fish diet frequency per month of residents in 139 cities of China (kg).

In the past, especially 20 years ago when China’s highway network was not built, and food freezing technology was not developed, sea fish were seldom transported inland. Because sea fish will quickly die and deteriorate after leaving seawater, they were almost exclusively eaten by coastal-area residents. Even today, most sea fish in China are consumed by coastal area residents. There is no accurate per capita sea fish consumption data for Chinese cities, so we launched an investigation of the frequency of sea fish consumption, per month, for the residents of 139 cities in China, as a representative sample of the whole country. It would be too difficult to obtain this data from all cities, so the 139 selected cities account for 40.3% of all cities in China, and are distributed throughout every part of the country, as illustrated in Figure 12. Frequency of sea fish consumption is related to distance from the sea. In coastal cities, residents eat sea fish 3–10 times per month, while in inland areas most consume sea fish fewer than one time per month. We evaluated the consumption of sea fish for each city according the city’s per capita sea fish consumption and seawater food production in coastal regions (Equation (2)):

(2) $$C_{i}=\sum_{i=1}^{n}F_{i}\times \frac{T_{i}\times P_{i}}{\sum_{i=1}^{n}(T_{i}\times P_{i})}$$

In this equation, *C_i_* is per capita sea fish consumption in city *i*, *F_i_* is sea fish production in city *i*, *T_i_* is the monthly frequency of sea fish consumption in city *i*, and *P_i_* is the population of city *i*.

### 2.4. Methods

Kolmogorov-Smirnov tests were performed to determine whether the distribution for each of the factors above was normal. For those factors that were non-normal, logarithmic transformations were used to normalize the distributions. Spearman’s rank correlation was used to evaluate the relationship between each factor and the longevity ratio. A stepwise multiple linear regression (MLR) analysis and geographically weighted regression (GWR) were used to identify the factors significantly associated with the longevity ratio.

## 3. Results

Figure 1 and Figure 2 show that individuals living in southern and eastern coastal regions in China have greater longevity. Longevity is lower in northern regions, which feature lower humidity, higher monthly mean temperature standard deviation, and lower Se. Spearman’s rank correlation was used to evaluate the relationship between age and element concentration (Table 2). Positive correlations were seen between longevity ratio and sea fish consumption, humidity degree, T-Se content in soil, WS-Se content in soil, and freshwater food consumption, while negative correlations were seen for longevity ratio and altitude, monthly mean temperature standard deviation and meat consumption. However, meat consumption was not statistically as significant as the other seven factors. A regression analysis of these factors and longevity calculated associated correlations, as illustrated in Table 3.

Table 3 shows correlation coefficients for the nine factors in the regression model. Sea fish consumption, humidity, T-Se, altitude, and standard deviation of monthly mean temperature were more closely related to longevity than WS-Se, fresh water food consumption, or meat consumption. *p* values associated with meat consumption were higher than 0.05 in 2000 and 0.01 in 2010, indicating that neither factor was significant. No R^2^ was higher than 0.4, indicating that longevity is not determined by one single factor, and that a multiple factor analysis was necessary. Stepwise MLR was used to ascertain the association between factors and longevity ratio, Table 4 and Table 5 represent the stepwise MLR developed using longevity ratio as the dependent variable and environment and nutrition factors as the independent variables in 2000 and 2010, the R^2^ is 0.535 and 0.543, respectively. The final three independent variables of the model were standard deviation of monthly mean temperature, altitude and per capita sea fish consumption, both in 2000 and 2010.

After modeling, the classical multiple regression model fit was always lower than 0.6, and the Durbin-Watson value was 1.268. This showed that the residual of ordinary least square (OLS) has a positive autocorrelation structure and was not suitable for establishing models with OLS. The regression parameters in this study varied relative to geographic location. Regression parameter estimation of a global regression model is the average value of the regression parameters in a study area. This fails to reflect the spatial character of all the regression parameters. Factors in this study such as Se, temperature, humidity, altitude and fish consumption vary according to regional characteristics and spatial effects. In this case, we adopted a geographically weighted regression (GWR) model. In GWR, geographic location was embedded in the regression parameters. Consequently, observational data close to the position has more influence than data far from the position, resulting in adjacent locations having similar regression parameters. We tested all the factor groups and found that the correlation coefficient of the group consisting of humidity, altitude, and per capita sea fish consumption was highest, both in 2000 and 2010. In addition, the top four groups with highest R^2^ are illustrated in Table 6 and Table 7.

In 2000 and 2010, regression coefficient of humidity, altitude and per capita sea fish consumption were always higher than groups made up of other factors, which indicates that the three factors are most related to longevity at the city scale. Regression coefficient of WS-Se, altitude and per capita sea fish consumption were always ranking the second place.

By comparing Table 4 and Table 6, and by comparing Table 5 and Table 7, the correlation coefficient of each group significantly improved (higher than the multiple liner regression model) in the GWR model. The predicted regression value of longevity by GWR with humidity, altitude, and per capita sea fish consumption, for each city, are illustrated in Figure 2. 

**Figure 13:**
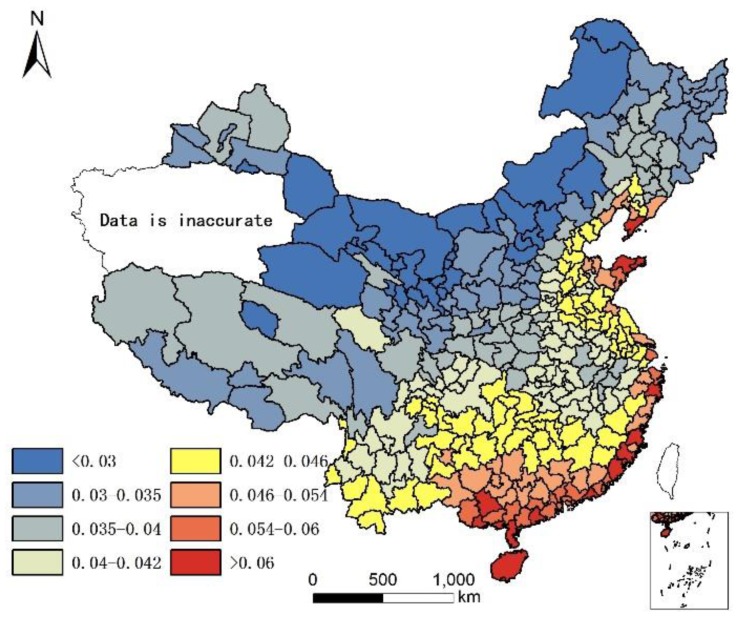
Simulated Longevity rate (85+/65+) in China with altitude, per capita sea fish consumption and humidity in 2000.

**Figure 14:**
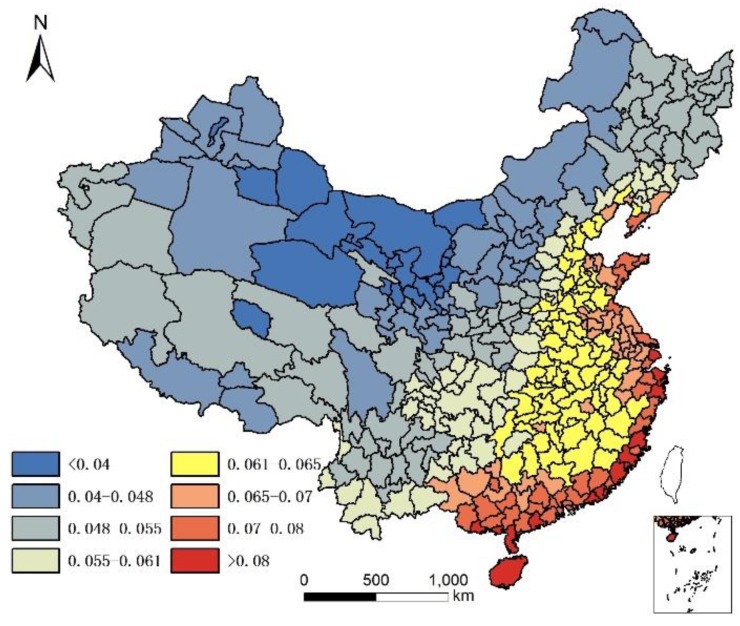
Simulated Longevity rate (85+/65+) in China with altitude, per capita sea fish consumption and humidity in 2010.

**Figure 15:**
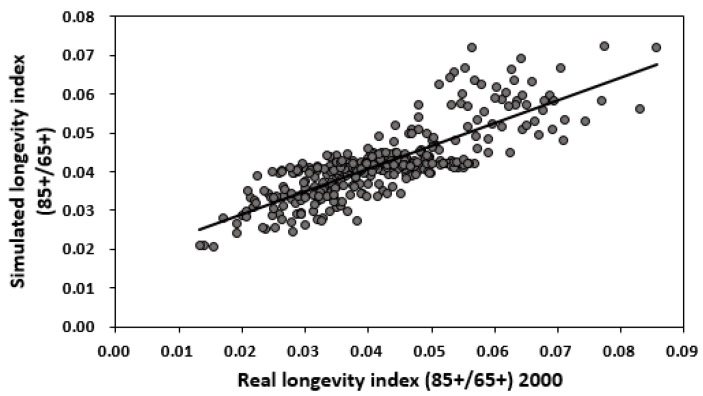
Relation between real longevity index and simulated longevity index by humidity content in soil, altitude and per capita sea fish consumption in 2000.

**Figure 16:**
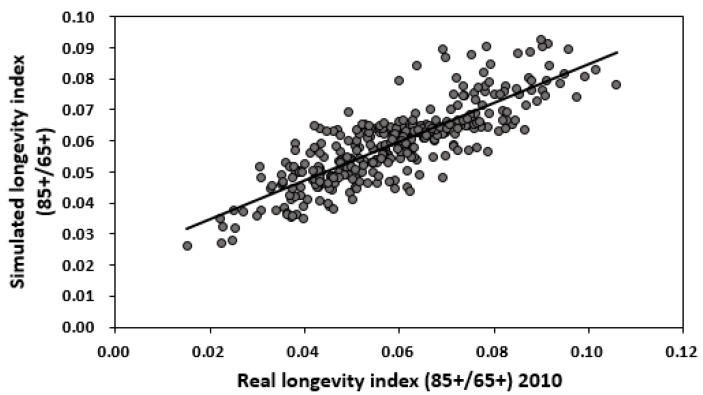
Relation between real longevity index and simulated longevity index by humidity content in soil, altitude and per capita sea fish consumption in 2010.

By comparing Figure 13 and Figure 1, and by comparing Figure 14 and Figure 2, we observe a good fit with most places in China, especially in the middle and eastern regions. The relation between the real longevity index and the simulated longevity index is illustrated in Figure 15 and Figure 16.

## 4. Discussion

The nutrition factors are crucial to maximizing the longevity ratio, particularly the consumption of the trace element Se, and sea fish. Meat consumption was not related to the longevity ratio. Se is an essential trace element to humans, and average Se contents in the body decrease with increasing age. Se in hair decreased significantly through intergenerational transmission (grandchildren > children > centenarians) [26], which means Se supplementation is important for healthy aging. In this paper, the correlation coefficient of Se with the longevity ratio is not as high as temperature and humidity. This is because Se varied largely even at a small scale in soil, high Se content and low Se content usually coexist in a city, and the research scale of this paper is still too large to show relationship between Se and longevity. Some studies at the town scale and county scale within a city have shown that climate factors is almost the same, so Se will be more significant related to longevity [24,25,26,27,28].

**Figure 17:**
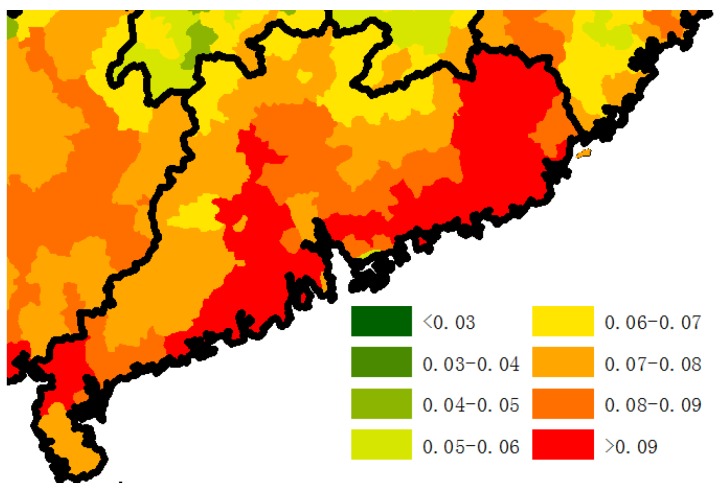
The 85+/65+ ratio of county scale in Guangdong province.

Sea fish consumption appears as an important factor in both stepwise MLR and GWR. The consumption of sea fish, kelp, seaweed, jellyfish, mussels, and other sea foods are excellent sources of protein and unsaturated fatty acids. They also supply inorganic salts, which hinder the absorption of cholesterol in the intestines, all of which are associated with healthy diets. Almost all the cities along the coast, whether developed or poor, had high longevity ratios. Although some coastal cities in China are richer, one-third of them remain below the average gross domestic product (GDP) for all cites in China, including Huludao, Dandong, Jinzhou, Yingkou in Liaoning province, Shanwei, Jieyang, Chaozhou, Zhanjiang, Shantou, Maoming in Guangdong province. A relatively low longevity ratio was found in the coastal regions of Hebei province, where the production of sea fishing was also lowest among the coastal regions. In Guangdong province, the longevity ratio gradually decreases moving inland from coastal regions, as illustrated in Figure 17.

Although high consumption of sea fish may lead to the health risks of metal exposure, a research on the concentrations of metals (Cd, Cr, Cu, Hg, Ni, Pb and Zn) of fishermen found that there was no health risk from most of the metals, because they did not exceed their related reference doses, even if the daily fish consumption of residents was as high as 283, 366 and 469 g/day in children, women of childbearing age and the remaining population groups, respectively [33]. Overall, sea fish consumption is positively related to longevity in China as long as the consumption is not too much.

The environment factors temperature, humidity, and altitude are tolerance factors that relate to longevity. In other words, a moderate climate is more suitable for a longer human life. In Figure 1, all of the cities with an 85+/65+ ratio > 0.07 (top quartile) were located in areas that were not very cold during winter. In fact, all these cities had an average temperature >10 °C except Dandong city (7.7 °C), and most were >15 °C (the average was 18 °C), had humid air (all with precipitation > 550 mm; average precipitation 1345 mm; humidity > 24), and low altitude (<1000 m except Leshan city (1131.68 m)). The average altitude of cities with superior longevity was 201 m. However, not all the cities with moderate climates ranked within the top quartile for the longevity index. One hundred fifty-five cities had a standard deviation of monthly mean temperature < 10, humidity > 30, and altitude < 1000 m; however, only 72 of them had an 85+/65+ ratio > 0.07. In addition, the cities with highest longevity ratios do not have the lowest altitudes, lowest monthly mean temperature standard deviations, or highest humidity. Therefore, there is no significant difference in longevity between different sub-climatic types within moderate climate.

**Figure 18:**
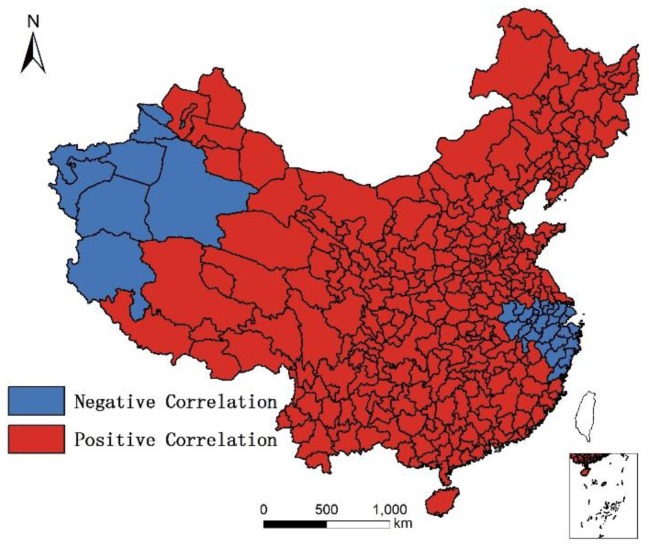
Distribution of negative/positive correlation between humidity and longevity.

**Figure 19:**
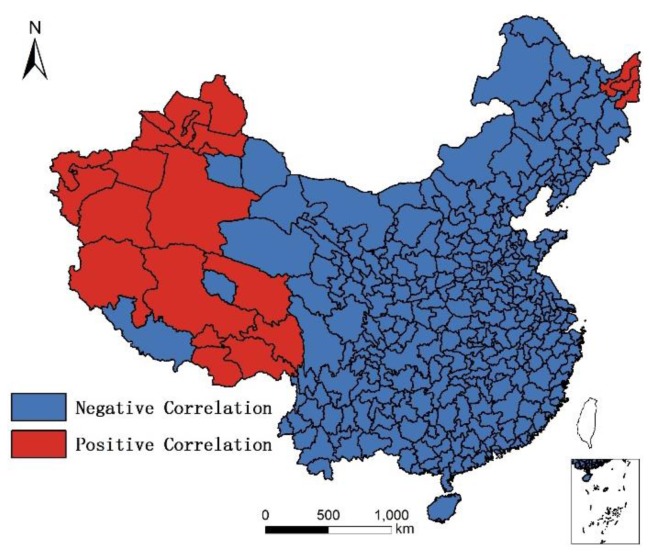
Distribution of negative/positive correlation between altitude and longevity.

In addition, although humidity is positively correlated with longevity, and altitude is negatively correlated with longevity, the results of non-linear regression in GWR shows that in the moistest place in Southeast China, humidity is negatively correlated with longevity, as illustrated in Figure 18; and in highest place in West China, altitude is positively correlated with longevity. The reason for these phenomenon is unknown, as illustrated in Figure 19.

There are also some limitations to this study: The reliability of census data in 2000 has been questioned, especially in Xinjiang [34]. Therefore, we deleted the data in south Xinjiang in 2000. There is no accurate per capita sea fish consumption index for each city in China. Consequently, we evaluated sea fish production based on survey results from only 139 cities. Meat and freshwater fish production were used as proxies for meat and freshwater fish consumption because there are no official per capita meat and freshwater fish consumption indexes. As previously discussed, assumptions about migration and pollution were also made which might mean key factors explaining the geographical distribution of longevity might be missing. What these limitations also point to is the overall limitation of carrying out retrospective cross-sectional analyses.

## 5. Conclusions

This study used data from the Chinese population census and several environment and nutritional factors to examining the relationships among longevity and environmental and nutritional factors. The main finds were:

1. The 85+/65+ ratio is higher in coastal and southern regions of China.
2. Climate factors, sea fish consumption and the Se content of soil are related to longevity, while meat, freshwater fish consumption are less related to longevity.
3. The regression parameters in this study varied relative to geographic location; geographically weighted regression is more suited to reflecting the spatial characteristics of regression parameters, compared to stepwise multiple linear regression. During geographically weighted regression, the correlation coefficients improved significantly.
4. The combination of the humidity, altitude, and per capita sea fish consumption explained 66% and 68% of longevity in China in 2000 and 2010, respectively.
5. Moderate climate is more conducive to longevity, however, there is no significant difference in longevity between different sub-climatic types within moderate climate, and the relation between humidity and longevity is not always positive, the relation between altitude and longevity is not always negative. Nutritional factors like omega-3 in sea fish are crucial to longevity.

Although our results can explain a great deal of general factors related to longevity, there remain inexplicable phenomena. For example, a minority of cities located in the Tibetan plateau also have a mid-range longevity ratio (the 85+/65+ ratio is between 0.06 and 0.07). Interestingly, the altitude there is >3000 m and many find the climate intolerable. Examples such as these warrant further investigation and discussion.

## Figures and Tables

**Table 1:** Water food production in China in 1990 and 2015 (10^4^ Ton).

Year	Seawater Food Fishing	Seawater Food Aquaculture	Freshwater Food Fishing	Freshwater Food Aquaculture
1990	551	162	79	446
2015	1315	1876	228	3062

**Table 2:** Spearman’s correlation coefficients for longevity ratio and each factor. Total selenium (T-Se); water-soluble selenium (WS-Se).

Year	Sea Fish	Humidity Degree	T-Se	Altitude	SD of Temperature	WS-Se	Freshwater Food	Meat
2000	0.575 **	0.468 **	0.505 **	−0.338 **	−0.502 **	0.401 **	0.123 *	−0.105 *
2010	0.551 **	0.535 **	0.466 **	−0.461**	−0.442 **	0.303 **	0.205 **	−0.129 *

** Correlation significant at *p* < 0.01 level; * Correlation significant at *p* < 0.05 level. “SD of temperature” represents standard deviation of monthly mean temperature.

**Table 3:** Linear regression coefficient of each factor with longevity in regression analysis.

Year	Sea Fish	Humidity Degree	T-Se	Altitude	SD of Temperature	WS-Se	Freshwater Food	Meat
2000	0.331 **	0.219 **	0.255 **	0.114 **	0.252 **	0.161 **	0.015 *	0.011
2010	0.304 **	0.287 **	0.217 **	0.212 **	0.195 **	0.092 **	0.042 **	0.017 *

** Correlation significant at *p* < 0.01 level; * Correlation significant at *p* < 0.05 level.

**Table 4:** Regression coefficient, standard error, t-value and probability of the estimated parameters in longevity model by the stepwise multiple linear regression (MLR) analysis in 2000.

Parameter	Standard Coefficient	Standard Error	T Value	*p* Level
Intercept		0.002	30.594	0.000 **
SD of temperature	−0.445	0.000	−10.986	0.000 **
Altitude	−0.310	0.000	−7.719	0.000 **
Sea fish consumption	0.360	0.000	8.662	0.000 **

T Value is the value of T-test; *p* Level is Probability Level; ** Correlation significant at *p* < 0.01 level.

**Table 5:** Regression coefficient, standard error, *t*-value and probability of the estimated parameters in longevity model by the stepwise MLR analysis in 2010.

Parameter	Standard Coefficient	Standard Error	T Value	*p* Level
Intercept		0.003	33.157	0.000 **
SD of temperature	−0.404	0.000	−10.136	0.000 **
Altitude	−0.429	0.000	−10.874	0.000 **
Sea fish consumption	0.302	0.000	7.338	0.000 **

** Correlation significant at *p* < 0.01 level.

**Table 6:** Regression coefficient of the top four groups with highest R^2^ (Regression coefficient) of geographically weighted regression (GWR) in 2000.

Group	R^2^	Group	R^2^
Humidity, altitude, sea fish	0.664 **	WS-Se, altitude, sea fish	0.641 **
SD of temperature, altitude, sea fish	0.623 **	SD of temperature, humidity, sea fish	0.619 **

** Correlation significant at *p* < 0.01 level.

**Table 7:** Regression coefficient of the top four groups with highest R^2^ of GWR in 2010.

Group	R^2^	Group	R^2^
Humidity, altitude, sea fish	0.681 **	WS-Se, altitude, sea fish	0.659 **
T-Se, altitude, sea fish	0.639 **	SD of temperature, altitude, sea fish	0.628 **

** Correlation significant at *p* < 0.01 level.
